# The Evolution From Standardized to Virtual Patients in Medical Education

**DOI:** 10.7759/cureus.71224

**Published:** 2024-10-10

**Authors:** Allan Hamilton, Allyson Molzahn, Kyle McLemore

**Affiliations:** 1 Surgery, University of Arizona, Tucson, USA; 2 Arizona Simulation Technology and Education Center, University of Arizona, Tucson, USA; 3 Artificial Intelligence Division in Simulation, Education, and Training, University of Arizona, Tucson, USA

**Keywords:** holography, humanoid animatronics, immersive reality, medical education, standardized patient, virtual reality, virtual standardized patient

## Abstract

Standardized patients (SPs) are widely used in medical education to teach clinical skills and provide assessments. SPs allow students to practice history taking, physical exams, and communication in controlled settings. However, SPs have limitations such as fatigue, performance variability, and the inability to simulate certain conditions, which virtual patients (VPs) can address. VPs can address these limitations and offer consistency, scalability, and adaptability. Although VPs are being implemented in research settings, they have the potential to be powerful medical education tools. Advancements in immersive technologies such as virtual reality, haptic feedback, and artificial intelligence (AI) will allow the creation of hyper-realistic, interactive training environments that mimic the complexity of real patient encounters. Medical students will be able to engage with VPs in fully immersive settings, complete with haptic feedback and AI-driven dialogue, allowing for more lifelike diagnostic and procedural experiences. The wider availability of such technologies through web services has implications for global medical education and assessment.

## Introduction and background

Medical education has long relied on standardized patients as the primary medium for medical students to practice the patient encounter. However, the COVID-19 pandemic required remote patient encounters for medical student training, shifting attention toward enhancing virtual patient platforms. With advancing technologies, virtual patients offer consistency, adaptability, and scalability, positioning them as the future of medical training.

## Review

The advent of standardized patients

Since the major medical educational reforms promulgated by the release of the Flexner Report in 1910, there has been a great emphasis on the accessibility of patients in both ambulatory and in-patient settings for the education of medical students and other healthcare professionals [1]. However, over the course of a century, the availability of such patients as training material has declined for both economic and ethical reasons [2]. Increasingly, the position has been that the justifications for employing patients for training purposes are and should be sharply constrained. The introduction of medical simulation-based training has not only largely supplemented the use of actual patients for clinical training but also largely supplanted using them for procedural training [3]. As the movement for instituting medical simulation as a mainstream method of enhancing clinical exposure and acquiring procedural proficiency, there was an increased need to introduce skilled human actors to portray simulated patients and further enrich the opportunities for patient-care provider communication [4].

The introduction of standardized patients (SPs) represented a new use of trained individuals who enacted the role of a patient solely for training purposes. The use of SPs for teaching and evaluating clinical medical skills was first carried out by Howard S. Barrows at the Los Angeles County Hospital [4]. He initially labeled the use of a healthy individual for this purpose as a “pseudo patient” or a ”teaching patient.” However, later they became known as a standardized patient. This definition included both actual patients trained to present their illness in a standardized way and simulated patients without the illness but trained to simulate it in a standardized way. He discovered one of the strengths of individuals who had become educated about the illness they were elected to portray as an SP was they could assure the training director that the portrayal of the condition could take place with a high level of clinical accuracy and set the stage for a complete work-up of the patient’s history, symptoms, and also even how certain physical findings might be elicited.

Geoff Norman, a psychometrician from McMaster University, eventually coined the term “standardized patient” because he felt it helped underline the major advantage of this clinical examination technique, namely, providing a patient with a particular set of clinical problems that would not vary from student to student [5]. Norman studied a library of illnesses to develop an encyclopedic list of findings suitable for simulation and acquisition by accomplished SPs. Later in his career, Norman became a faculty member at the Southern Illinois University (SIU) School of Medicine, where he developed a rotating multi-station, multi-SP layout where students would have a 15-20-minute encounter with an SP before proceeding to the next station to work up an SP with a new clinical problem.

Many of the features of the SIU approach would later be incorporated into the Objective Structured Clinical Examination (OSCE). With the support of the Joshua Macy Jr. Foundation, the OSCE eventually became incorporated into the standard evaluation of medical students for proceeding to more advanced training in the later years of medical school [6]. It proved to be a more effective and objective assessment of students’ abilities and, by offering multiple SP stations, a more exhaustive one [7].

The standardized patient in medical education and training

SPs have been proven effective in a wide variety of applications that range from situations like training students how to communicate bad news to families under different circumstances, determining the quality of care at the point of delivery in the field by other providers, refining interpersonal communication skills, and enhancing early stroke intervention in SPs presenting to first responders [8-11]. However, there are also drawbacks to using SPs. These include the individuals serving as SPs suffering from performance anxiety, fatigue, and other indicators of generalized “burnout” [12]. The use of SPs has been shown to introduce sources of variance and errors. Any assessment method must be evaluated and reconsidered over time to look for many of these same sources of variable reliability. SPs have shown inconsistency from station to station (that involve the issue of content specificity in the medical-problem-solving literature and may be related to the relatively long serial examination period), show greater consistency in pass/fail formats as opposed to mastery-testing approaches, enhanced reliability when used in combination with checklists to create standardized milestones on clinical examination, and inter-rater and inter-group variability [13]. However, there has been a good correlation between positive test results from SP examination exercises and other rating systems of clinical proficiency [14]. Like many educational and assessment methodologies widely deployed across hundreds of medical schools, there can be glaring inconsistencies found when careful comparisons reveal profound differences in methodologies across different locales, personnel, and types of practices [15].

SPs have become an integral part of the medical education landscape. Only eight of 195 medical schools in the United States did not advertise the use of SPs in their teaching programs to prospective applicants [16]. Market forces have combined to substantively increase the population of SPs utilized in medical education. These have included expanded use and applicability through the United States Medical Licensing Examination (USMLE) as administered by the National Board of Medical Examiners. In addition, there have been efforts to duplicate the use of SPs by the Educational Commission For Foreign Medical Graduates to provide standardized reports that allow for quality assurance and documentation for foreign medical graduates seeking entry into programs in the United States [17]. Simultaneously, there have been several medical school initiatives and multiple supervising organizations that have created pilots to help develop standards for the training of SPs and the development of case materials for application to SPs [4].

How far can we go with standardized patients?

Nonetheless, there have been efforts within independent medical schools to begin developing standards for quality assurance evaluations and standardized training for SPs, but these efforts have been variable to date. Although SPs rarely make significant amounts of money for their contributions, discussions continue about the economic impact of SPs on educational and assessment budgets, especially in light of the rising utilization of SPs [17]. The need for ongoing audits and direct supervision of SP programs has been reinforced as it helps to identify deficits as well as to enhance student feedback and standardization of case administration. Nonetheless, compared to other techniques widely used in medical student education, such as clinical vignettes in written clinical summaries, SPs remain the gold standard [18].

However, several theoretical concerns have been brought up for discussion about the extensive use of SPs in today’s healthcare education curricula. First, economic issues arise over ever-broadening applications for SPs and the financial burden they place on medical schools. More importantly, concerns have been raised about the physical discomfort and fatigue of the SPs themselves, particularly when they may be called upon to play multiple scenarios for several hours. Although the literature does not suggest there are negative health effects of being an SP, there have been theoretical concerns raised about the long-term physical and emotional effects of being asked on a regular and sustained basis to present themselves as somebody who is ill. Some SPs report changes in their own perceptions of healthcare and healthcare providers, largely stemming from a loss of confidence in primary medical care due to the nature of the scenarios and incidents that arise during portrayals [12]. In addition, not all patient populations are as amenable to being portrayed with SPs as others. For example, children and adolescents have been engaged as SPs, but there are ethical concerns, particularly for children in the younger age groups [19].

From hybrid to virtual patients

SPs are not suitable for scenarios where invasive procedures are required. The need for a simulated patient that could withstand significant physical manipulation (e.g., cardiopulmonary resuscitation) requires high-fidelity mannequins. However, the mannequins have their shortcomings. They cannot converse, so a coherent history had to be provided by a technician or clinician. Recently, there has been a tendency toward an increased utilization of so-called “hybrid simulations.” The term has applied to a confusing array of technologies and approaches. For example, the term hybrid simulation has been applied to situations where an actor is outfitted with wearable technology. Such an individual can obviously converse freely and accurately report symptoms, but also exhibit limited vital signs. This approach has been recommended for institutions that might not be able to afford the cost of buying and maintaining high-fidelity manikins [20]. However, the same designation of “hybrid simulation” has been applied to situations where an SP plays the actual human patient, interacts with the medical team, and is simply accompanied by a mannequin in the room. This allows the team to carry out invasive procedures on the mannequin as part of the scenario [21]. Finally, there has been a trend toward utilizing more advanced equipment to provide pertinent physical examination findings. For example, when a case scenario with an SP includes the findings of a 3/6 systolic ejection murmur on auscultation, an MP3 file of the murmur can be substituted in a so-called multi-purpose simulation stethoscope [22]. Prerecorded heart sounds are then played into the stethoscope, triggered by the placement of the simulation stethoscope in proximity to radio frequency identification (RFID) tags. Other models use Wi-Fi capability to alter sounds heard in the simulation stethoscope in real time for the benefit of the examinee [17,23]. One clear-cut advantage of SPs is the emphasis on high-quality patient-provider communication [24].

Essentially, two factors have played into the development of virtual patients (VPs). The first was the COVID-19 pandemic, which put enormous pressure on health education facilities to develop remote methods whereby a patient could be evaluated. This occurred in combination with significant improvements in telehealth technology and equipment, which made it easier to process large numbers of students through remote evaluation [25]. In the wake of the pandemic, as educational institutions gradually recovered some modicum of normality, it became clear that large-scale SP evaluations could occur with reduced costs because SPs no longer had to travel to a centralized location. Video recording at a distance also became more feasible, while the newer video conferencing systems provided adequate protections to meet the requirements of both the Health Insurance Portability and Accountability Act (HIPAA) of 1996 and the Family Educational Rights and Privacy Act (FERPA) of 1974 [26].

Virtual patients

In the 1990s, scholars like L.J. Whalley at the University of Aberdeen began to pose both technical and ethical questions about how emerging technology and virtual reality (VR) would be applied in medicine. Theorists felt sure that the first advances in VR would be employed for modeling complex pathophysiologic regulatory systems. In such situations, extensive databases would be combined with functional mapping of organ function, and these could serve as helpful laboratory models to study disease. The second predictable application of VR was introducing it into environments in which patients would be included. This would involve, for example, the monitoring of anesthesia or the reading out of ICU monitoring equipment in a VR environment [27]. In addition, VR can be used to provide a virtual contactless interface in the operating room (such as a keypad) where a surgeon might need to enter data at multiple intervals without contamination. Finally, introducing supercomputers allowed more exquisite modeling to quantify some of the relationships between therapeutic interventions and outcomes [28]. The final stage would be envisioned as each patient would be empowered to enter their own data directly into the model. In essence, the real patient becomes subsumed into the virtual endeavor [29].

In 1999, step three of the USMLE allowed for the inclusion of VPs [30]. Not only was this a major administrative and institutional hurdle to overcome, but it was also clear that economic forces and biomedical advancement were transforming the assessment of proficiency in healthcare. For example, the pace at which patients pass through the hospital system often disrupts the continuity of care between multiple services and teams. This meant there was an increased demand to provide opportunities where trainees could evaluate a patient throughout the full course of their illness and subsequent recovery - something that was becoming more difficult to mirror clinically [31].

In the late 1990s and 2000s, a VP was defined as “a computer program that simulates real-life clinical scenarios in which the learner acts as a healthcare professional obtaining a history and physical exam and making diagnostic and therapeutic decisions” [30]. One crucial distinguishing characteristic of VPs was they could more easily simulate the longitudinal course of a particular problem. This gave medical students a feel for the evolution of a chronic illness over a long interval - something that would not be feasible with the SP [32].

Several research studies demonstrated VPs were well received by trainees and, in some cases, students might even refine certain skill sets better than they had with SPs. For example, medical students who were using VPs to learn about maladies such as acute back pain rated the learning experience as more enjoyable and performed better on standardized assessments than students who had read medical literature on the topic [33]. But, other concerns surfaced. For example, did something happen to a student’s moral compass when patients ceased being individuals with whom one met at appointed times, in appropriate settings, under finite circumstances? There was the potential for patients to be increasingly visualized as computerized chimaeras, spun out of thin air at will, to be intercepted for a full interview, or a period of minutes, that their physical exam might be started or never finished. One student explained that a “VP was a patient to whom you could do absolutely anything…that you wanted …” [34].

As educators astutely pointed out, VPs come in essentially two categories: problem-solving and narrative [35]. The two states are not mutually exclusive but formative. The objective sets in motion a certain intention that betrays design. The problem-solving VP is a species that ideally excels at providing interconnected facts and findings that reliably lead the student (armed with a modicum of knowledge and imagination) through a body of material and information related to a particular class of illness and its specific pathophysiological manifestations. One could justifiably call the problem-solving VP an immersive extension of curricular materials.

By contrast, the narrative VP is a different animal with a different design. Here, there is little sense that one set of cues neatly leads the learner to the next set of data points in a predictable fashion. The narrative VP (sometimes referred to as a “systematic VP”) does not clearly allude to what the next course of action should be. Instead, the narrative VP is, like real human patients, capable of producing a profusion of information, both of a historical and/or physical nature. Amid this potential maelstrom of data, the discerning trainee is asked to navigate through a fog of historical facts and a body of symptoms, to lay out a course aligned by the pertinent positives and negatives, and then safely guide the patient to a diagnostic destination. The trick in the narrative VP is that it is clueless; it lacks any ability to “cue or clue” the student. The student must rely on an increasingly astute sense of recognition by constellations and aggregates of symptoms and findings [36].

In an attempt to better segregate the qualities and outcomes of these two types of VPs, researchers took simple cases (e.g., acute abdomen) and encapsulated them into a narrative design of a VP. The moderately simple case of abdominal pain could offer several different responses over a variety of time courses depending on the direction the student steered the interview. The problem-oriented VP provided far more cues and clues than in the narrative format. From an educational point of view, it was natural for a third-year medical student to feel more relaxed when conducting an interview where they were not confronted by an infinite number of answers. The students found it easier to ask questions of the guided, problem-oriented VP than the narrative VP. After these two types of VP experiences, students noted that they had gained significant insights into the implicit and explicit role played by the physician in the interview [37]. Other researchers evaluated the impact of altering the VP's affability and willingness to answer questions and demonstrated that diagnostic accuracy exhibited by the medical students fell as the VPs were perceived as being “less friendly” [38]. Students felt they were being asked to play more of a “doctor-expert” role when they were working with the narrative design format of the VP than if they were involved in the problem-solving format. Students expressed greater satisfaction with the problem-oriented simulation because they felt they were actually allowed to make choices while the narrative scenario involved more of the emotional responsiveness of the patient. Students reported the narrative style of VPs as an opportunity to develop a better rapport and working relationship as the interview progressed. Interestingly enough, medical students also felt they provided more satisfaction to the VP in the narrative format [39].

The value of this kind of research is difficult to underestimate. We often think of the VP as essentially a passive object simply because it has been created by a computer and software package. However, the interactivity of that avatar with the medical student has significant implications both directly with problem-solving, but also indirectly with the degree of emotional satisfaction the student derives in serving the patient in the capacity as physician. Finally, by introducing a wider range of emotional variables and allowing the students to engage the VP longitudinally over the course of several encounters, the encounters reinforced the importance of deriving an emotionally gratifying relationship with the VP [40].

Scenarios for VPs are usually created by educators and are based on templates of pre-existing cases. Recently, there have also been attempts to create algorithms for branching cases where students may select one of several options during the stage of a case. This would be more akin to a viewer being able to select how a movie ends [41]. In addition, there are now student-authored VP cases that are based on games with different students playing different roles in the interaction [42]. What is not clear is exactly how reliably feedback and reporting are taking place with the different VP designs. Nor have there been extensive attempts to explore the validity and fidelity of the VP scenarios with reference to their real-life transferability [43]. It is recognized that this could present substantial difficulties in orchestrating such a VP project, but in the end, the whole point of simulation is that it is transferable to the care of real patients. Finally, there are also models of VPs where the facilitator educator can take into consideration the cognitive load the student can reasonably carry [44].

VPs and artificial intelligence (AI)

Educators typically shape scenarios for VPs based on the templates of pre-existing cases. In essence, the VP (like the SP) functions like an actor who is called upon to follow a script. However, with the introduction of AI, we can look to a future where AI is building the VP based on the students’ needs. With AI, the VP can be custom-tuned to the problem-solving abilities of the trainee. The difficulty of the patient’s presentation, course, and management can be adjusted to impose a cognitive load commensurate with the student’s fund of knowledge and clinical experience [45]. AI’s facility for developing sustained storylines for VPs allows one to create scenarios where an individual student can intercept the same VP at different points in the course of their illness or the same illness at different stages of the life cycle or even follow several alternative courses (e.g., complication) [46]. This solves one of the big problems that students are encountering with a lack of continuity and patient contact given the rapid turnover rates in clinic and in-patient facilities [47].

With this proliferation of VP models, there is concern that a lack of standardization could adversely affect the validity of the historical narratives and symptoms that the patients are reporting. How reliable and classic are the physical findings? Do these physical findings represent a valid constellation of positive and negative findings so that we are not just teaching the students a narrow perception of how diseases manifest themselves [48]? While the lack of standardization has been a problem in the past, we are seeing an increasing confluence of AI, particularly with the use of large language models (LLMs). Given that LLMs can accommodate a wide variety of clinical history, presentation, diversity of patients, and cultural contexts, we believe there will be an enhanced rationale within the simulation community to tackle the issues of validation [49]. Finally, one of the challenging assignments remaining will be to calibrate the interaction between the facilitator, the VP, and the medical student. It is one thing to have a VP and an AI program provide feedback on a medical student’s performance, but it is quite another to reframe a healthy and productive coaching relationship between faculty and students in that context [50].

Manufacturing VPs in the Age of AI

The core fabrication of VPs can be separated into four categories: (1) VP construction, (2) external preconditions, (3) student-VP interactivity, and (4) consequences. The VP construction refers to the materials an educator or facilitator has integrated into the fabrication of case scenarios. It will also include Interactive elements that allow a student to take a patient history, conduct an examination, order tests, and make clinical decisions. Increasingly, there is a need for a library to quickly pertinent clinically positive or negative findings, and a user-friendly computer interface (e.g., “dashboards”) to permit data to flow into the case as needed [51,52].

External preconditions affect the student and instruction-centered factors. This includes the manner in which positive and negative experiences arise in the course of completing the scenario. Preconditions also include following up on whether the specific learning objectives of the VP scenario were met. At an organizational level, the aims of the curriculum and evaluation of students’ experience are vital areas and make significant contributions to what has been labeled “the institutional fingerprint” with respect to the expectations of the students as they approach VP-driven educational material [50,53,54].

Student-VP interactivity refers to how VPs and trainees interface. Much of this refers to the style of the interaction, specifically the questioning style and type of engagement between the learner and VP. Included in this may be unexpected consequences. For example, a student might become “bogged down” in relatively irrelevant information, while overlooking explicit positive and negative findings. This may include concerns about false expectations or conclusions based on what the student did or did not see during the course of the scenario [51]. Much of this will be directly attributable to the VP constructed in terms of directly providing feedback versus providing it to the faculty for a post-scenario debriefing [55].

The final element of VP construction is the consequences, and this is meant to ensure that educators attend to the after-effects that students may have experienced after interacting with the VP. Special attention needs to be paid to items that might affect the behavior of the student in future practice. Evaluation should also include developing a plan to address specific weaknesses. There is a wide variation in how students experience handling a case scenario with a VP. A significant factor seems to rest on the students’ prior experience with VPs and their “buy-in” to the actual scenario design [52].

VPs in immersive virtual reality (IVR)

In IVR, participants interact within a virtual scene or environment from an egocentric point of view (POV). The participant may be able to visualize themselves from an out-of-body POV or see just their hands, for example), within the virtual space. The key to grasping the IVR is that the participant is wearing goggles (and often ear “buds” or headphones) and there is no reference to an external environment. Individuals interact within the ”immersive depiction” by means of a tracking device, which is responsible for the participants' movements and locomotion within the IVR. IVR with multiple participants usually requires sufficient bandwidth and computing power that made them so expensive that they were generally reserved for industrial or military training regimens. In the last five years, the cost of immersive equipment has fallen substantially (especially because of the demands of the gaming industry) and IVR team training is now feasible. IVR scenarios often involve individuals being assigned roles on a team that may be tending to a VP is a particular scenario to evaluate interprofessional team skills and leadership [56,57].

The future of virtual patients

There has been a significant increase in the capabilities of VPs. The future of VPs in medical education will be shaped by advances in AI, haptic technologies, and robotic patient simulators. With rapid advances in video and audio capabilities of AI-driven VPs, a virtually limitless catalog of human patients and conditions will rapidly become available for medical student use. The breadth and subtlety of symptoms and signs will expand the capacity of AI-designed VPs, so encounters are custom-tailored to a student’s level of education sophistication and procedural proficiency [58].

VPs in the Age of AI

Prior to the application of LLMs to VPs, each response from an avatar or a high-fidelity manikin (HFM) had to be entered manually from a keyboard or pre-recorded responses were essential. This approach often produced gaps in the ability of the VP to adequately address reasoning skills from the VP and resulted in poor and often halting, stilted dialogue that interfered with the immersive quality of a learning experience. With the advent of generative large language multi-modal models (GLLMMs), the value of VPs in medical training has been dramatically enhanced.

Grant et al. relate how the isolation imposed by the COVID-19 pandemic led to the accelerated development of suitable VPs to meet trainee demands [59]. Under pandemic conditions, a typical OSCE-like exchange between a student and an SP encounter was essentially downgraded to a conversation between the two on a 2D display. This incentivized the development of an early “patchwork” version of a VP by assembling images, MP3 (audio), and MP4 (video) files. Students were also able to augment images in real time using an interactive whiteboard.

Within one year’s time, a mixed-reality application for OSCEs was developed that combined VPs in an immersive virtual learning environment. Using a design-based research approach, an app was developed in 2022 on the Microsoft Power Platform (MPP; Microsoft Corporation, Redmond, WA). The MPP was a relatively low-cost platform with a collection of low-code development tools that permitted animation. Dynamic content with reasonably realistic VPs was constructed on the MPP using the MetaHuman Plugin for Unreal Engine (MHPI; Epic Games, Cary, NC). MHPI allowed a VP coder to develop a custom “mesh” of the human body or face and then animate it. While the project aimed to enhance virtual clinical examinations by allowing students to interact with the VP via a pen scribe to indicate examination landmarks, it also explored new opportunities in VP representation, animation, and interactivity [60,61].

Visual Realism

Visual realism has a considerable impact on the level of engagement of participants. Visual realism can be broken down into two components. The first is geometric realism which describes how much the virtual object looks like the real object. The second is realistic illumination, which refers to the fidelity of the lighting as it falls on the object in question [62]. In fact, both geometric and illumination realism have dynamic aspects to them and need to be well-tuned to look realistic. Some computer systems use a static lighting system applying a method called radiosity, which pre-computes diffuse interreflections in the environment [63,64]. Many IVR systems use neural radiance fields (NeRFs), which is a method that uses deep learning to reconstruct a three-dimensional representation of a scene that is derived from sparse two-dimensional images. As a rule, highly realistic VPs that are able to exhibit skin mottling, appropriate emotional facial reactions, or facial droop concomitant with a cerebrovascular accident are greeted enthusiastically by learners [65]. More recently, the introduction of 3D Gaussian splatting (3DGS) replaces the traditional polygonal mesh depiction of the body with a collection of 3D Gaussian particles (called “splats”) that allows for real-time rendering of complex, photorealistic movement (often at speeds approaching 100+ frames per second, which is much faster than other techniques like NeRFs). Although admittedly, realism in VPs is a moving target with the rapid pace of improvements in AI [66].

With respect to the potential of creating hyper-realistic, interactive holographic representation, an initiative was undertaken to record the experiences of Holocaust survivors as the number of eye-witness survivors is diminishing with increasing age. A technically sophisticated audiovisual project was undertaken to reliably produce a series of 3D interactive holographic images that would be able to permanently sustain the personal presence of the holocaust survivor as an immersive, conversational interaction, long after the actual individual had expired [67]. Realistic holograms have successfully been produced using generative AI to create immersive and interactive holographic displays of Holocaust survivors that can interact in real time with an interviewer [68]. This technology shows potential for application to VPs and pilot evaluations show that students demonstrate a high level of engagement with 3D holographic representations [69].

Haptic Technologies in Virtual Patient Encounters

Students must learn physical exam maneuvers such as palpation, auscultation, and percussion and interpret the findings in the context of the patient’s presentation [70]. Despite their many advantages, VPs suffer from a lack of physical interaction that is crucial to medical training. They cannot replicate the tactile feedback and hands-on experience that students gain from working with SPs. Palpation-related skills are challenging to teach because they require precise hand movements, pressure application, and real-time physical feedback that can only be experienced through direct interaction. These subtle tactile clues and manual dexterity are difficult to convey through current virtual interfaces.

Currently, physical exam findings may be requested by the student and provided as a video or written summary [71]. However, this bypasses the crucial step of the student conducting and interpreting the physical exam. A number of approaches have been attempted to circumvent the palpation issue. These include wearable human-machine interfaces, such as smart gloves, which allow medical students to conduct the physical exam with a VP as they would with a real patient [72]. However, the incorporation of realistic haptic interactions in VPs still remains elusive. This is a significant shortcoming since tactile communication is an important part of building a healthcare provider and creating a trusting relationship with a patient [73]. While haptic technologies are advancing significantly to improve the sensitivity and granularity of the feedback, there are significant barriers to integrating this into AR/VR experiences. Overcoming these barriers will contribute to a more realistic and effective VP experience for students.

Humanoid Robotic Patient Simulators (HRPSs)

Currently, high-fidelity medical simulation mannequins constitute one of the most common devices used to simulate human beings. SPs obviously are real human beings acting as patients suffering from medical illness. HRPSs would be expected to be independently animated and capable of many (if not all) human movements. In addition, they would be expected to have a wide range of expressivity to permit them to demonstrate powerful emotions, neurologic impairment, as well as states of discomfort, illness, or pain [74]. The capacity to project real-time facial expressivity requires a complicated robotic framework underlying the face along with the appropriate synthetic materials that would move appropriately to computer commands [75]. There are centers that are at work on facial expressivity and animatronic robotic simulators have been developed for such applications as movies. For the time being, the animatronic technology along with the microprocessors required to move limbs in a coordinated fashion is still very expensive and difficult to maintain. However, we remain confident that it is only a matter of time before a VP can be replaced by a high-fidelity HRPS. They will be capable of providing feedback, including all pertinent modalities such as sight, hearing, palpation, and percussion [76].

## Conclusions

Several discrete venues of technological advancements will lead to effective and realistic VP experiences. There are several considerations regarding implementing VPs into the medical education curriculum. For one, the initial cost of both VP and robotic patient simulators may not produce reasonable returns on investment for some programs. Once a system to support VPs is in place, it could offer a wealth of patient experiences that are completely customizable. VPs can be deployed on-demand, allowing for broader reach and consistent training experiences. The scalability of VPs can also offer an ideal solution for institutions with limited resources or in more isolated communities. A great deal of work lies ahead in the validation of VPs, scenario training, assessment, curricular integration, and the impact of VPs on the faculty-student interaction and, finally, on whether VPs will represent the next giant technological leap forward or, merely, an expensive sideshow.
